# A Challenge

**Published:** 1915-01

**Authors:** 


### A Challenge.

Apropos of this growing sentiment for prevention, the *Purity Journals* asks: "How long will it take to reform the world, if we work only at *cure* and ignore *prevention?*" Quoting further, the *Journal* says: "The progress of the race has been slow, largely because what has been gained in one generation has been lost in the next. Not only has the vast fortune of the pirate of one age been dissipated by his children; but the far richer treasures of the wise have not been handed down to their posterity. Man has chained the lightning and made it his servant; he has transformed the desert into a fruitful garden; he has produced from the delicate wild rose the rich American beauty; from the unsavory crab, the delicious Baldwin; from the untamed wolf, the intelligent St. Bernard; from the useless equidae, the thoroughbred draft horse; from the ordinary cow, the priceless Alderney; from the barefoot boy, born in poverty and reared in privation, the greatest man that ever filled the presidential chair, if not greater than any man that has occupied the throne of kings.

"But these incidents, which are the result of careful design in improving flowers, fruits and the lower orders of creation, are in the human race almost universally the result of unconscious, involuntary observance of pre-natal laws. Instead, therefore, of human beings taking advantage of these beneficent laws, they almost altogether ignore them. Consequently, the improvement and uplift which should be universal, is found only in rarest cases and then only partially developed.

"The Universe is governed by law and not by chance. Certain causes produce certain effects. Man has recognized this truth in every sphere except that of character. For ages the best efforts of the race have been devoted to overcoming the tendencies to evil rather than in trying to produce good tendencies. The work of transformation is glorious; but it is only one phase of the truth. Formative work is equally important and, when it comes to results, it is as superior as gold is to gilt. * * *

"The power of parents to produce character in their children prenatally; that is, before birth, is one of the greatest, most important facts of the universe. If we are to have better children, we must first have better parents. In prenatal influences it is not profession, but practice, that counts. It is not the outward life, only, but the heart life. It is absolutely impossible to deceive nature. She is impartial and does not swerve a hair's breadth because the person is a king or potentate or is an inhabitant of the slums. * * *

"The observance of these truths will rapidly bring humanity up to its highest possibilities. It will speedily transform the systems of the world from selfishness to the rule of love and true philanthropy. It will eliminate injustice which now handicaps race progress, and will make true happiness possible. It will remove the need for prisons, almshouses and asylums. It will bring to the earth true civilization in fact as well as in name. It will cause justice to take the place of injustice. Righteousness to reign in the place of vice."

